# Effect of Albumin Polymorphism on Thyroid Hormones: A Case Report and Literature Review

**DOI:** 10.7759/cureus.2903

**Published:** 2018-07-01

**Authors:** Rupak Mahendhar, Amir Shahbaz, Maria Riaz, Michael Aninyei, David M Reich, Issac Sachmechi

**Affiliations:** 1 Internal Medicine, Icahn School of Medicine, Mount Sinai/Queens Hospital Center, New York, USA; 2 Internal Medicine, Icahn School of Medicine at Mount Sinai/Queen Hospital Center, New York, USA; 3 Internal Medicine, Icahn School of Medicine at Mount Sinai/Queens Hospital Center, New York, USA; 4 Geriatrics, James H, Johnson City, USA; 5 Medicine, Icahn School of Medicine, Jamaica, USA

**Keywords:** familial dysalbuminemic hyperthyroxinemia, human serum albumin, thyroxine, arginine

## Abstract

Familial dysalbuminemic hyperthyroxinemia (FDH) is the most common cause of the inherited increase of serum thyroxine in Caucasians. This disorder occurs due to a missense mutation in the human serum albumin, resulting in an increased affinity of thyroxine to the binding sites on the human serum albumin (HSA) molecule. HSA is a carrier protein of thyroid hormones and only 10% of thyroxine (T4) is bound to human serum albumin, 75% is bound to thyroxine-binding globulin, 15% to transthyretin, and 0.03% is free. The disorder is characterized by a greater elevation of serum thyroxine than triiodothyronine (T3). The high serum concentration of T4 is due to the modification of a binding site located in the N-terminal half of HSA (in subdomain IIA). Arg218 or Arg222 gets replaced with smaller amino acids, such as histidine, proline, or serine, due to missense mutation; this reduces the steric hindrances in the binding site and creates a high-affinity binding site for thyroxine. We herein report a case of FDH with a characteristically elevated total T4 and normal free T4 (measured by equilibrium dialysis).

## Introduction

Thyroid hormones (TH) (T3 and T4) circulate in the blood bound to the carrier proteins, i.e., thyroxine-binding globulin (TBG), transthyretin (TTR), prealbumin, and human serum albumin (HSA). Thyroxine-binding globulin, which has the highest affinity for thyroxine (T4) (Kd = 0.1 nM), binds about three-quarters of the hormone carried in the circulation; the remainder is divided more or less equally between transthyretin and HSA. Albumin binds T4 with a Kd of ≈2 μM and provides an important fast-response reservoir for the hormone during capillary transit [1]. Extracellular T4 and T3 are protein-bound by 99.97% and 99.7%, respectively. Because free thyroid hormones exist in equilibrium with the carrier proteins, any factor that affects the quantity/quality of the carrier invariably affects the pharmacokinetics and pharmacodynamics of the thyroid hormones [2]. This is best exemplified in familial dysalbuminemic hyperthyroxinemia (FDH), an autosomal dominant disorder characterized by an elevated total T4 (TT4), elevated free T4 (FT4), normal thyroid stimulating hormone (TSH), and T3 level, as measured by a one/two-step immunoassay. Some rare cases of elevated T3 have been described. We describe the case of a patient with FDH presenting with hyperthyroxinemia.

## Case presentation

A 79-year-old Hispanic male with a history of hypertension, schizoaffective disorder, benign prostatic hypertrophy, and chronic renal insufficiency was admitted for evaluation of a wide complex tachycardia found on his outpatient 24-hour Holter monitor done to evaluate his complaint of frequent palpitations. The patient acknowledged that he had palpitations for several years and that he was always nervous. He also stated that this “nervousness runs in the family”. There was no associated weight loss, unusual hair loss, or visual changes. He also denied any neck mass/goiter, diarrhea, or abdominal skin rash/thickening but stated that his hand always “shakes”. He denied any chest pain or shortness of breath, either at rest or with exertion. He also denied any orthopnea or paroxysmal nocturnal dyspnea but had complained of chronic leg swelling with no recent change. Exercise tolerance remained very good and unchanged.

On admission, an endocrinology consult was requested for evaluation of persistently elevated TT4 and FT4 levels. His vital signs were stable and physical exam revealed a mildly anxious elderly male with coarse tremors of the hands on extension. The rest of the examination was unremarkable. A sonogram of the thyroid gland showed bilateral prominent lobes, the left greater than the right. A thyroid iodine (I)-123 uptake scan was 19.4% (normal level: 15 - 40%). It also showed a mildly enlarged but non-palpable lower pole of the thyroid lobe with homogenous activity throughout the gland. Thyroid function tests (TFTs) done using routine automated direct one-step/two-step immunoassays showed normal TSH levels of 1.22 and 0.78, respectively (normal level: 0.34 - 5.6 mU/mL). T3 levels were normal at 98.4 and 125.3 ng/dL (normal level: 87 - 178 ng/dL). However, TT4 and FT4 levels were elevated. FT4 levels were 2.25 and 2.34 ng/dL (normal level: 0.58 - 1.64); and TT4 levels were 17.58 and 18.69 (normal level: 6.09 - 12.2). Free T3 by equilibrium dialysis was 402 ng/dL (normal level: 230 - 420) and an alpha subunit for pituitary hormone was less than 0.3 ng/ml (normal level: < 1.0). Free T4 by equilibrium dialysis was normal at 2.1 ng/dl (normal level: 0.8 - 2.7).

Cardiac workup, including echocardiogram, cardiac enzymes, stress test, and telemetry monitoring, was unremarkable. He was treated with a beta-blocker and discharged after being assured that his thyroid condition was benign.

## Discussion

Familial dysalbuminemic hyperthyroxinemia (FDH) is an autosomal dominant inherited disorder identified in 1979 independently by Henneman et al. [3] and Lee et al. [4], and it was not until 1994 that Petersen [5] and Sunthornthepvarakul [6], independent of each other, identified the precise genetic defect. FDH is characterized by a missense mutation in the human serum albumin, which is a protein synthesized by the liver. Of the several iodothyronine-binding sites on the HSA molecule, only one has a relatively high affinity for T4 and T3. A mutation on this binding site leads to higher concentration of thyroid hormone in the bloodstream. The mutation involves codon 218, which is normally arginine, and it gets replaced with histidine, proline, or serine (or codon 222), which is also arginine that gets replaced with isoleucine. These mutations in the codon 218 and codon 222 for a smaller amino acid reduces the steric hindrances and creates a high-affinity binding site for T4 [2, 9]. Individuals with FDH are heterozygous for the mutation. Although they may present with altered thyroid hormone levels, they are clinically euthyroid.

Table 1 describes the various albumin variants, the mutations causing these variants, its prevalence, and also the factors by which the concentration of thyroid hormones are increased.

**Table 1:** Albumin Variant and Its Mutations HSA: human serum albumin; TFT: thyroid function tests; FDH-T4: familial dysalbuminemic hyperthyroxinemia thyroxine; TT4: total thyroxine; TT3: total triiodothyronine; rT3: reverse triiodothyronine; UL: upper limit

HSA TYPE	ALBUMIN MUTATION	TFT	PREVALENT POPULATION
R218H (FDH-T4)	Guanine to adenine mutation in the second nucleotide of the codon for Arg218 [2].	TT4: ↑ 1.1 - 1.8 times the UL; TT3: ↑ 0.6 - 1.2 times the UL; rT3: ↑ 0.7 - 1.4 times the UL	North America, Western Europe, Eastern Asia, Hispanic, and New Zealand.
R218P (FDH-T4)	Guanine to cytosine mutation in the same nucleotide of Arg218 [2].	TT4: ↑ 8 - 15 times the UL; TT3: ↑ 1.2 - 2.1 times the UL	Japan (Aomori prefecture)
R218S (FDH-T4)	Cytosine to adenine mutation in the first nucleotide of the codon for Arg218 [2].	TT4: ↑ 7 times the UL; TT3: ↑1.6 times the UL; rT3: ↑ 2.4 times the UL	Found in one Canadian family.
R218C (FDH-T4)	Cytosine to thymine mutation in the first nucleotide [2].		This variant has not been reported yet.
R222I (FDH-T4)	Guanine to thymine in the second nucleotide of Arg222 [7].	TT4: ↑ 1.3 - 2.0 times the UL; rT3: ↑ 40 - 70 times the UL	Found in 1 Croatian family and 3 unrelated families of East Africa origin.
Leu66 (FDH-T3)	Thymine substituted by cytosine in the second nucleotide of codon 66 [2, 8].	TT3: ↑ 1.4 times the UL; TT4: ↑ 0.7 times the UL	Thai

Our case report and TFT results fit the classic description of FDH, an autosomal dominant disorder characterized by an elevated TT4, elevated FT4 (as measured by one-step/two-step immunoassay but normal by equilibrium dialysis), a normal or rarely elevated T3, and normal TSH. The estimated prevalence of FDH among the Caucasian population is 1 in 10,000 individuals, and it is much higher in subjects of Hispanic origin, i.e., 1.0 - 1.8% [2].

The initial total and free T4 assays were performed using the labeled antibody method. This is the common method of choice for initial testing in most centers due to the wide availability and low cost. To confirm or better delineate an abnormal result, direct equilibrium dialysis is the next test of choice, as it was in our patient. While this method has a better ability to accurately report true free T4 values in the face of confounding variables, false-positives may still occur [9]. Hoshikawa et al. have reported false-positive results of elevated free T4 assays using equilibrium dialysis/radioimmunoassay (RIA), and they suggest that this assay is not an ultimate standard for diagnosing FDH, especially in patients with the R218P mutation of the ALB gene [10]. They further recommended ultrafiltration as an added measure to increase the accuracy of testing prior to RIA.

On review of the literature, we found the following studies and case reports (shown in Table 2) regarding familial dysalbuminemic hyperthyroxinemia.

**Table 2:** Review of the Literature FDH: familial dysalbuminemic hyperthyroxinemia; FT4: free thyroxine; HSA: human serum albumin; pH - potential of hydrogen; RIA: radioimmunoassay; T3: triiodothyronine; T4: thyroxine; TBG: thyroxine-binding globulin; TSH: thyroid-stimulating hormone

AUTHORS	STUDY INTRODUCTION	STUDY SUMMARY
Choudhary et al. [9]	This is a case report of a 4-year-old girl with signs and symptoms attributable to hyperthyroidism who had discordant thyroid test results. Genetic analysis confirmed FDH. Equilibrium dialysis was done as a gold standard assay to confirm FDH but showed false-positive results.	This is a unique case of FDH where equilibrium dialysis resulted in a false-positive FT4. This case highlights the need for a combination of familial and genetic testing, as well as a close scrutiny of the results obtained, even with gold standard assays.
Hoshikawa et al. [10]	This is a case report of a pregnant Japanese woman with FDH caused by mutant albumin R218P. She had elevated total T4 levels and normal FT4.	In this patient, T4 binding to albumin was increased during pregnancy and decreased postpartum. Her FT4 was elevated when measured with equilibrium dialysis but not by ultrafiltration. The study demonstrated that the equilibrium dialysis/RIA was not the ultimate test and ultrafiltration followed by RIA was better for diagnosing FDH in patients with albumin variant R218P.
Cho et al. [11]	A case report of FDH with an albumin variant Arg242His in a Korean family.	The Arg242His albumin variant resulted in a mild form of FDH that produced HSA with enhanced affinity for T4 (10- to 15-fold increase) and resulted in high T4 levels with a two-fold increase.
Bianchi et al. [12]	This study was done to investigate the role of thyroxine-binding globulin (TBG) and albumin in the availability of thyroid hormones to peripheral tissues.	The study indicates that T4 bound to FDH albumin-binding sites, although less available than T4 bound to normal albumin, is more available to peripheral tissues than T4 carried by TBG.
Sachmechi et al. [13]	This is a case report of a pregnant Puerto Rican woman who was recognized to have FDH during her pregnancy.	This study recommends that FT4 measured by the one-step method should be corroborated with equilibrium dialysis; even the diagnosis of thyrotoxicosis is highly likely due to a suppressed TSH level. The study also showed that a normal FT4 level after equilibrium dialysis makes FDH as the most likely diagnosis, ruling out TSH-secreting pituitary adenoma and thyroid hormone resistance syndrome.
Stockigt et al. [14]	The study is about the response of pituitary-thyroid axis and thyroxine-binding to plasma proteins in three kindreds with familial euthyroid thyroxine excess.	It suggested that the thyroxine excess in these affected subjects following treatment with exogenous T4 or T3 was an appropriate response to abnormal T4-binding so as to maintain normal T4.
Divino et al. [15]	This study was done to demonstrate the role of pH and anionic binding inhibitors in increasing T4-binding affinity to the variant albumin of FDH.	The findings from the study suggested that an electrostatic bond between iodothyronine phenolate and a cationic group on the protein was the basis for the increased affinity and specificity of variant albumin for T4.
Dughi et al. [16]	In this study, fluorescence was used to find out conformational changes between normal HSA and FDH-HSA.	The study hypothesized that the FDH-HSA solution contains an equimolar mixture of FDH-HSA and normal HSA. The solution has a weaker component and stronger component binding site. While the weaker component represents normal HSA T4-binding site, the stronger component represents the FDH-HSA T4-binding site.
Stockigt et al. [17]	The study has analyzed a method of free T4 measurement [Amerlex® Free T4(Amersham International, UK)] using a novel unidentified T4-analogue together with an antibody that binds both T4 and analogue.	The study demonstrated discrepancies in the assay used to measure free T4 due to variations in binding of the T4-analogue with binding proteins and came up with an assumption that equilibrium dialysis gives the best estimate of free T4.
Rajathanavin et al. [18]	The study assessed thyroxine analogue radioimmunoassay method used to measure free thyroxine level in euthyroid patients with FDH.	The study suggested that 125I-labeled T4 derivative cannot be used to correctly diagnose patients with familial dysalbuminemic hyperthyroxinemia as it showed an elevated FT4 level due to direct binding of the 125I-labeled T4 derivative to the abnormal serum albumin that binds T4.

Some of the important differential diagnoses that need to be considered as well include TSH-secreting pituitary adenoma, a rare condition accounting for 2% of all pituitary adenomas, which is characterized by elevated/normal serum TSH and elevated TH levels with high alpha subunit. The patient is usually thyrotoxic, and magnetic resonance imaging (MRI) usually shows a pituitary tumor/hyperplasia [19]. Thyroid hormone resistance syndrome is another important differential, characterized clinically by goiter, sinus tachycardia, and elevated/normal serum levels of TSH and elevated thyroid hormones: thyroxine (T4) and triiodothyronine (T3). It is rare and inherited in an autosomal dominant fashion. The primary defect in a thyroid hormone-resistant syndrome patient is usually a mutation involving the thyroid hormone receptor beta, and recently, mutations in the thyroid hormone receptor alpha gene have been identified which are associated with near-normal thyroid hormone levels with hypothyroid features [20]. Therefore, a high clinical suspicion of FDH is required in evaluating euthyroid patients with elevated total T4 and normal FT4 levels. In order to avoid unwanted treatment of euthyroid persons with hyperthyroxinemia or hypertriiodothyroninemia, protein sequencing and/or sequencing of the albumin gene should be performed.

## Conclusions

Familial dysalbuminemic hyperthyroxinemia is usually benign and does not usually require treatment unless there is a concurrent different thyroid dysfunction. Because of the paucity of knowledge of this condition, particularly among non-endocrinologists, there is a tendency for inappropriate treatment, including radioactive iodine, medications, and thyroid surgery and hence, the need for extensive education on this condition.
